# Delivering an evidence-based outdoor journey intervention to people with stroke: Barriers and enablers experienced by community rehabilitation teams

**DOI:** 10.1186/1472-6963-10-18

**Published:** 2010-01-19

**Authors:** Annie McCluskey, Sandy Middleton

**Affiliations:** 1 Community-Based Health Care Research Unit, Faculty of Health Sciences, The University of Sydney, New South Wales, Australia; 2 National Centre for Clinical Outcomes Research (NaCCOR), Nursing and Midwifery, The Australian Catholic University, Australia; 3 Nursing Research Institute, St Vincent's and Mater Health Sydney and the Australian Catholic University, New South Wales, Australia

## Abstract

**Background:**

Transferring knowledge from research into practice can be challenging, partly because the process involves a change in attitudes, roles and behaviour by individuals and teams. Helping teams to identify then target potential barriers may aid the knowledge transfer process. The aim of this study was to identify barriers and enablers, as perceived by allied health professionals, to delivering an evidence-based (Level 1) outdoor journey intervention for people with stroke.

**Methods:**

A qualitative design and semi-structured interviews were used. Allied health professionals (n = 13) from two community rehabilitation teams were interviewed, before and after receiving feedback from a medical record audit and attending a training workshop. Interviews allowed participants to identify potential and actual barriers, as well as enablers to delivering the intervention. Qualitative data were analysed using theoretical domains described by Michie and colleagues.

**Results:**

Two barriers to delivery of the intervention were the social influence of people with stroke and their family, and professionals' beliefs about their capabilities. Other barriers included professionals' knowledge and skills, their role identity, availability of resources, whether professionals remembered to provide the intervention, and how they felt about delivering the intervention. Enablers to delivering the intervention included a belief that they could deliver the intervention, a willingness to expand and share professional roles, procedures that reminded them what to do, and feeling good about helping people with stroke to participate.

**Conclusions:**

This study represents one step in the quality improvement process. The interviews encouraged reflection by staff. We obtained valuable data which have been used to plan behaviour change interventions addressing identified barriers. Our methods may assist other researchers who need to design similar behaviour change interventions.

## Background

### Translating Evidence into Practice

Translating evidence into practice, or implementation is an active process involving individuals, teams and organisations [1]. Knowledge translation is an important final step in the process of evidence-based practice. This step is challenging and involves changes in attitude and behaviour. Researchers cannot assume that an intervention which demonstrates a positive effect and has been described in a high impact journal will be translated in practice [2]. Nor should researchers assume that the majority of people with a health condition will receive that intervention [3].

Barrier identification is an important first step in the process of knowledge translation [4]. Failure to anticipate problems and barriers may lead to disappointing results [1]. Barriers to knowledge translation include limited skills and knowledge, negative or outdated attitudes, unhelpful procedures and role identity problems [5]. Some of these barriers may also be enablers. For example, a senior health administrator may reject a practice change or act as an opinion leader and promote change [6]. While it is possible to anticipate some barriers, assumptions should not be made about which barriers affect a team or health service [7]. Nor should it be assumed that barriers will be the same for two apparently similar services.

### Translating Evidence into Community Stroke Rehabilitation

In community stroke rehabilitation, there is evidence that mobility and transport training can improve health outcomes for people with stroke. However anecdotal reports suggested that this evidence was not often being used to guide practice in our local region. Evidence from a systematic review of exercise programs, mostly delivered by a physiotherapist, highlighted gains in walking speed and distance following task-specific training [8]. Mean improvements in speed (0.14 metres/second) and distance (41 metres on the 6-Minute Walk test) were reported following gait-orientated training across 21 trials. One trial involved community-dwelling people with stroke who received four weeks of treadmill training and overground walking practice [9]. In that trial, walking capacity increased by 86 metres (95% CI 44 to 128) more than for controls, with speed gains being maintained after three months.

Local physiotherapists in our region were providing treadmill training and overground walking practice, but most of the intervention was conducted in the hospital gym. Few sessions were conducted outdoors. Even fewer sessions involved visiting the streets and suburbs where people lived. Yet people with stroke in New Zealand have reported an ongoing loss of confidence on ramps, escalators and in shopping malls even after several weeks of physiotherapy [10]. In that study, improved walking indoors did not improve walking outdoors. To gain confidence and skills, people with stroke seem to need community-based training in context.

There is also evidence that escorted journeys with a therapist can improve participation and the quality of life post-stroke [11]. However, again, anecdotal reports indicated that this evidence and escorted outdoor journeys were rarely being delivered to people with stroke. In that Level 1 trial, a typical program involved up to seven sessions with an occupational therapist to increase confidence crossing roads, negotiating shopping malls and/or when catching a bus (median of 6 sessions); information was also provided about local transport services and driving [12]. People with stroke who received this outdoor journey intervention got out twice as often, and doubled the number of outdoor journeys taken per month, compared to controls. Gains were maintained after 10 months. The outdoor journey intervention is recommended as best practice in Australian national clinical guidelines for stroke rehabilitation [13]. However, an evidence-practice gap was known to exist in Sydney, Australia where the authors were located.

### The Evidence-Practice Gap

To help identify how much this intervention was being used in practice, we conducted a medical record audit (n = 77) in 2007 with five community rehabilitation teams [14]. The audit revealed that only 17% of people with stroke had received six or more sessions to increase outdoor journeys and mobility from an occupational therapist, physiotherapist or therapy assistant. Occupational therapists rarely escorted people with stroke to the shops or on buses. Physiotherapists rarely practiced road crossings or pavement kerbs outdoors. If therapists were to translate the evidence-based outdoor journey intervention into practice, we first needed to determine what they knew about the evidence (knowledge), if they felt the evidence was strong enough to justify a change in practice (attitudes and intentions), and how capable they felt about delivering the intervention in practice (skills and capabilities).

### Aims of the Study

The aim of this study was to identify barriers and enablers, as perceived by allied health professionals, to delivering an outdoor journey intervention to people with stroke, an intervention which had previously been shown to improve outcomes in a Level 1 trial [11,12]. This knowledge could then be used to inform an education program, to translate evidence into practice.

## Methods

A qualitative paradigm and study design were used to explore experiences, attitudes, knowledge and behaviour and reasons for the evidence-practice gap. The primary method of data collection was semi-structured interviews. Participants were allied health professionals from two community teams in Sydney, Australia. Ethical approval was obtained from a local area health service (Ref No. 2007/019) and a university ethics committee (Ref No. 10092).

As with many funded projects, we had a pre-determined aim to address in a relatively short timeframe, and qualitative findings had to link with quantitative audit data. Therefore, a framework approach was used [15,16]. This type of qualitative methodology is often used in applied or policy research where timescales are limited. The approach was first described by researchers at the National Centre for Social Research in Britain [17]. Data are explored inductively, and reflect the original stories or accounts of participants (that is, the findings are 'grounded' in the data) [16]. The framework approach also starts deductively with pre-set aims, and is more structured than other qualitative paradigms [16].

The five stages of the framework approach [16] are: familiarisation with the raw data; identifying a framework based on a priori aims and the raw data; developing an index of themes and concepts using representative quotations; producing a chart or table containing distilled summaries of views and experiences from several participants; then mapping and interpreting phenomena, creating typologies, comparing, and seeking associations between, themes to help explain the findings. The final interpretive stage has many similarities with constant comparative methods as used in grounded theory [18] and in small-scale studies [19] during analysis. These stages and methods are described in the following pages.

### The Sample

Two teams were selected purposively. Purposive sampling ensured that two different models of service delivery were represented: a domiciliary team that mostly conducted home visits (Team A), and an out-patient team that mostly saw people with stroke on-site at the hospital (Team B). Both teams were located in suburban Sydney, Australia and had offices in the same building. Professionals within these two teams were then invited to participate. While five services participated in the medical record audits and feedback sessions, only two teams were invited to participate in the barrier identification research project, due to limited funding.

Teams A and B both accepted rehabilitation referrals for people with stroke, multiple sclerosis and other neurological conditions, people with lower limb amputations, and older people with fractures secondary to a fall. Team A accepted referrals for people aged 65 years and over who had rehabilitation goals immediately post-discharge. They provided two or three rehabilitation sessions per week per person, for 12 week post-discharge, in the client's home. Team B accepted referrals for people of all ages and a range of diagnoses. Team B typically provided one therapy session per week per person, for six weeks, almost always based at the hospital. All rehabilitation services were provided free to people with stroke as part of Medicare, the publicly-funded health system operating across Australia.

All 13 allied health professionals employed across the two teams were invited to participate, were recruited and interviewed. Written informed consent was obtained from each person, immediately before their individual interview. Most of the 13 participants (88%) were occupational therapists or physiotherapists. Participant demographics are presented in Table 1.

**Table 1:** Demographic characteristics of allied health professionals (n = 13)

Characteristic	N	%
**Discipline**		
Occupational therapist	6	(45.2)
Physiotherapist	3	(23.2)
Speech pathologist	2	(15.3)
Other (Therapy assistant; social worker)	2	(15.3)
		
**Primary Work Role**		
Clinician - No Supervisory Role	9	(69.4)
Senior Clinician - Supervisory Role	2	(15.3)
Team Coordinator/Service Manager	2	(15.3)
		
**Timing of Interviews**		
Pre-Implementation Training (mid 2007)	11	(84.6)
During Implementation (mid 2008)	8	(45.2)#
		
**Number of Interviews**		
One	7	(53.8)
Two	6	(45.2)
		
**Employment Status**		
Full-time	10	(76.9)
Part-time (≤ 25 hrs per week)	3	(23.1)

Note. # Proportions do not add up to 100% because of staff changes. Of the 11 staff interviewed pre-implementation training, three had resigned (n = 1), rotated to another area (n = 1) or taken maternity leave (n = 1) by mid 2008. Of the eight staff interviewed in mid 2008, two were new employees.

### Data Collection

Interviews were conducted once or twice with each participant, at the hospital where both teams were based (some participants left before the second interview, while other new staff joined at the time of the second interview). The first author (AM) who conducted all interviews was an occupational therapist and health services researcher, with 30 years of clinical experience mostly in stroke rehabilitation. She was not employed by the area health service, and did not work on a day to day basis with any of the participants or teams. Most of the team members being interviewed knew the interviewer personally or by reputation, because of her research background. As the researcher's role and position could influence what participants said, they were advised that judgements would not be made about they said or what they knew.

Interviews were audiotaped with permission, then transcribed. First interviews were conducted in mid-2007, prior to implementation of the evidence-based outdoor journey intervention. Second interviews were conducted in mid-2008, after feedback from a medical record audit had been provided and a half-day training workshop delivered. By the second interview, most team members had been delivering the evidence-based outdoor journey intervention for at least six months.

#### The Implementation Training Workshop

The half day workshop was lead by the first author. First, we presented a critical appraisal of the original randomised trial by Logan and colleagues [11], and a description of the complex outdoor journey intervention [12]. Second, baseline audit data were presented with the permission of team members. Third, written protocols were presented for upgrading walking, bus and train travel training, trialling motorised scooters and consulting about return to driving. These protocols were prepared by the first author with advice from team members. Fourth, two case studies were presented by participating occupational therapy team members, describing the process of delivering outdoor journeys, goals of the person with stroke, treatment progression and safety tips. Finally, potential barriers to delivering the outdoor journeys were identified and discussed in teams. Examples of barriers and quotes from the initial qualitative interviews with team members were presented first. Participants were also asked to suggest possible solutions and enablers.

### Interview Questions and Procedures

Interview questions were developed by the first author. At the first interview, questions were designed to elicit responses about factors that might help or hinder the uptake of the outdoor journey intervention, in particular escorted journeys. After describing their discipline, years since graduation, years spent working with people following stroke and in the community, participating health professionals were asked to describe (1) what they knew about the outdoor journey intervention including the published evidence; (2) factors which might help or hinder their team from implementing the outdoor journey intervention. Prompt questions were used to enquire about skills and knowledge, staffing, physical resources, assessment, screening and report writing systems and treatment routines. Interviews took up to one hour. No particular theory or framework was used to guide data collection in the first round of interviews.

At the second interview 12 months later, similar questions were asked of continuing and recently employed staff. Participants were also asked to describe (1) people with stroke on their current caseload who were, and were not, receiving the outdoor journey intervention, and reasons for these intervention decisions (2) examples of recent therapy sessions where they and their team had provided intervention to increase outdoor journeys; and (3) factors that made it easy or difficult to provide the outdoor journey intervention.

### Data Analysis

Analysis began after the first interview had been transcribed and continued over 12 months [18]. Statements made by participants were interpreted then pasted into a computer file. Each category was given a provisional label such as 'skills' and 'resources'. The first three transcripts were independently analysed by AM and a research assistant who then met to reach consensus [20]. Coding was subsequently completed by the research assistant who met with AM every two to three weeks to review categories. In framework analysis, this stage is known as familiarisation [16].

Consensus was achieved during the early meetings by going back and forth between quotes and categories, and discussing the meaning of statements. Often a quote was moved from one category to another. For example, consensus was needed about the following quote *'...Carers might not have the confidence to take the patient out themselves....if they're less mobile and need assistance with transfers it would be difficult.... so convincing them to do it....that could be a hurdle...*.'. This quote was coded as a 'Skill' required by therapists (the skill of negotiating with family carers).

After the initial coding had been completed, we began using the 12 conceptual domains described by Michie and colleagues as the guiding conceptual framework or theory [5]. The process of identifying and applying a thematic framework represents the second stage of framework analysis [16]. The theory of behaviour change described by Michie and colleagues is intended for use by researchers who are exploring behaviour change, particularly barriers to evidence implementation. We selected this theory over others, including the theory of planned behaviour [21,22] and diffusions of innovation [23,24] because our initial codes and categories already matched several of the domains described by Michie and colleagues. For example, our early categories included 'Knowledge', 'Skills', 'Roles', and 'Influences'. Statements were initially allocated to one or more category. The statement '*Scooters are something that I would never bring up with a patient. If they do bring it up...I'm not sure what to do' *was first placed in two categories: 'Knowledge' and a new category 'Beliefs about Capabilities'. Another statement '*We wouldn't normally think to refer to occupational therapy for this aspect of care' *was also placed in two categories: 'Roles' and 'Memory and Attention'. The process of indexing data against an indentified framework equates to the third stages of framework analysis [16].

Tables or charts were generated, containing distilled summaries of participant views and experiences about barriers and enablers (a stage known as charting, the fourth stage of framework analysis). Statements were interpreted, compared with other participants' comments (constant comparison), then relationships examined across categories consistent with grounded theory methods of analysis [18]. This stage is also known as mapping and interpretation, in framework analysis [16], and was influenced by our original aims and objectives (to identify barriers and enablers which could be strategically targeted). We do not believe that use of a guiding theory and framework limited our analysis, since these were introduced after initial coding had been completed. Use of a theory of behaviour change and framework analysis [16], helped to streamline what can be a slow and costly process.

Previous interviews and ongoing analysis influenced the probing questions asked of participants, particularly during the second round of interviews. For example, we asked additional probing questions about 'factors' influencing the delivery of the outdoor journey intervention, questions about their perceived skills, knowledge, roles, and influences from people with stroke and their family. Analysis did not influence the number of team members interviewed (ie theoretical sampling was not used). All team members were invited to an interview and all participated. There was no attempt to achieve theoretical saturation.

## Results

Eight of the 13 participants were interviewed twice, and five participants were interviewed once only. Of the initial sample of 13, three had left their position before the second interview due to maternity leave (n = 1), staff rotation (n = 1) or resignation (n = 1). Two new team members were interviewed in 2008.

Factors that participants identified as potential or actual barriers to knowledge translation are presented below, followed by enablers. We first present quotes from the 2007 (baseline) interviews, followed by quotes from 2008 (follow-up) interviews. By 2008, participating team members had received feedback from a medical record audit, attended a training workshop where barriers and enablers were discussed, and some had delivered the outdoor journey intervention.

Two domains or categories, *Social Influences *and *Beliefs about Capabilities *were discussed often by participants as potential barriers to delivering the outdoor journey intervention (21 and 17 pages of quotes, respectively). These two domains will be discussed first, followed by five other barriers including *Knowledge and Skills*, *Resources*, *Professionals Role Identity*, *Memory and Attention *and *Emotions*. See Table 2 for a summary of the seven categories of barrier and sample quotations [see Additional file 1 for extra quotations about the barriers reported by participants].

**Table 2:** Barriers to delivering the outdo orjourney intervention with examples ^

**Social influences**: Unhelpful influences from people with stroke, family members, health professionals and community service providers that discouraged delivery of the outdoor journey intervention. Unhelpful influences included expectations about intervention, low tolerance for risk and restrictive organisational policies.

*From people with stroke: Some people are more focussed on getting their arm to work again 'I just want my arm to work'... [so] trying to identify goals different from that can be hard. Usually they've been told 'We're referring you for upper limb therapy' and they become very focussed on that 'I want to move my arm again'...they don't want to ....look at ...getting out and about. (OT1)*

**Belief about capabilities**: Unhelpful, pessimistic or ambivalent beliefs and attitudes about the capability of an individual professional, family member, or the team to provide the outdoor journey intervention.

*...there's concern about [therapy assistants] having the skills to assess and analyse the situation.....Would everything [ie problem areas] be picked up by somebody who's not trained? Can we have somebody who's not fully trained doing interventions and sessions? (OTM1)*

*Maybe [it's] partly to do with not having done this before...for people... gosh ...(thinks)...how do I get people on buses? And time wise.... [how do I manage]? (OT2)*

**Knowledge and skills**: Knowledge and skills which professionals felt they lacked, which related to; (i) the intervention itself; (ii) return to driving, (iii) motorised scooters and (iv) local transport options.

*One thing that the staff felt they wanted was information...around driving and what the guidelines say. Where can we access information to help them [people with stroke] for the knowledge part of the driving assessment? [Staff] awareness of what actually happens in an occupational therapy driving assessment is limited....how that's separate from the RTA [Roads and Traffic Authority] test, and sitting at a computer... and going out with a driving instructor? (OT3)*

**Professional role identity**: Unhelpful attitudes and beliefs about professional roles, and routines which presented a barrier to delivery of the intervention. Includes professionals who were pessimistic or ambivalent about role change.

*I think a lot more people COULD benefit from [this type of] occupational therapy than actually have it. That's the first barrier. We as non-occupational therapists wouldn't normally think to refer to [them] for this aspect of care.... much of the role seems to have been about upper limb training and maybe some cognitive training. (PT1)*

**Resources**: The absence of resources, limited resources or unpredictable resources on which the intervention depended. There were four categories of resources: (i) staff; (ii); time; (ii) information technology; and (iv) vehicles.

*Information technology: To get a bus timetable and transport information ...I'm lucky now I have access to the internet, but before I didn't, and you'd have to get someone else to log you on, which can make it difficult (OT1)*

*Vehicles: Getting cars has been a huge issue in the past. We don't have a [dedicated] car for that position. If there [wasn't] a car...it [would require] a bit more planning and approval (OTM1)*

**Memory and attention**: Failure or difficulty remembering to ask about, assess, document or address outdoor mobility and travel because of current systems, procedures or habits.

*Sometimes I might bring it [driving referral] up in discussion but I might forget when it comes time to document (OT1).*

**Emotions**: Negative or uncomfortable emotions when thinking about, or delivering the intervention.

*If I'm out with a client for three hours or so, there will be a feeling of guilt...it's a bit like a social outing...I know we're training community access but perhaps a feeling of guilt that you're gone for so long, with one person and you're out in the community (OT2)*

Notes. ^ The following abbreviations are used after quotations, to represent each profession: occupational therapy (OT); physiotherapy (PT); speech pathology (SP); and social work (SW). Numbers (eg OT1, PT2) correspond to de-identified individual professionals as summarised in Table 1

Enablers included changes in their attitudes, especially *Beliefs about Capabilities*, change in their *Professional Role Identity *(particularly role expansion), *Memory and Attention *(introducing procedures that reminded team members to act) and *Emotions *(positive feelings about the intervention). See Table 3 for a summary of the five categories of enabler and sample quotations.

**Table 3:** Enablers to delivering the outdoorjourney intervention with examples ^

**Social influences**: Helpful or positive influences from people with stroke, family members, health professionals and community service providers that encouraged delivery of the outdoor journey intervention. Influences included people expecting the intervention to be offered or provided, tolerance for risk and enabling organisational policies.

*From people with stroke: She's ...very motivated... keen to get back to catching the bus to the shops. She'll be good to work with. [On] my first visit last week, we walked up to the bus stop. She was lacking confidence... 'Are you sure it's not too early?'. I said 'No. It's not too early'*. (PT3)

*From policy: Our [community service] workers will take the person out if we specify 'The person can't go [shopping] on their own, so go with them. We want to encourage them [the person with stroke] to go. Take the patient with you'. (OT3)*

**Belief about capabilities**: Positive beliefs about individual abilities, and the ability of the team, to provide the intervention. Therapists' beliefs could change over time.

*Road crossing.....(long pause) I haven't really taken people across roads much at all. But we could do it...we could do it. We'd have to choose the roads...it's pretty scary out there [at the hospital entrance]...I take my life in my hands when I cross out there (laughs). (PT1)*

*[I was] thinking 'If someone has a heart condition, what would you do if they did collapse and you're on your own?'. They're the kind of things that go through your mind when you haven't done it. ... [so] it's helpful to have suggestions so that you're really prepared. So yeah, I've definitely started pre-thinking and trying to be as prepared as possible before going out (OT1)*

**Professional role identity**: Therapists who believed the intervention was compatible with, or could become part of, their professional role or the role of another team member. Having a positive attitude to role sharing and role expansion.

*In the initial interview we ask questions about ... shopping and banking and finances... how do they get there? Do they get public transport or drive? But beyond that, actually implementing anything is limited... [it is] something we should focus on (OT2)*

**Memory and attention**: Triggers and prompts that would help to remind therapists to think about, discuss and document outdoor mobility and travel.

*I think it should be in a questionnaire...that the OT and physio ask the client on [their] first meeting... More defined questions [like] " Do you want to go back to driving?" (OT2)*

*The [initial assessment] proforma that I'm using now goes into greater detail [about community access and transport] (OT5).*

**Emotions**: Therapists who felt positive emotions, including joy and satisfaction, from delivering the intervention.

*They [the therapists] enjoy it. [They say] 'I took them on the bus today and went to the shops'. (OT3)*

*They might be reluctant at first...but...you talk to them at the end and they [say] 'I know why I was pushed to do that' or 'If you weren't seeing me, I probably wouldn't have gotten to the stage that I am, trying to do this on my own'. Patients saying 'My confidence is so much better now, I don't think I would have got back to the bus if you didn't take me'. That's really ... rewarding. (PT3)*

Notes. ^ The following abbreviations are used after quotations, to represent each profession: occupational therapy (OT); physiotherapy (PT); speech pathology (SP); and social work (SW). Numbers (eg OT1, PT2) correspond to de-identified individual professionals as shown in Table 1.

### 1. Social Influences

The domain *Social Influences *refers to influences from people with stroke and family members, as well as health and community services. Any of these people or systems could encourage or discourage delivery of the outdoor journey intervention. Influences included expectations about intervention, tolerance for risk and organisational policies.

First, people with stroke influenced the interventions that therapists offered. Therapists reported that some people with stroke did not expect nor want to practice outdoor walking or travel with a therapist. Instead therapists felt they were expected to focus on interventions such as indoor walking, upper limb or self-care retraining.

Some people with stroke were afraid of falling, lacked confidence or experienced anxiety, which influenced what therapists did. One occupational therapist described helping a person with stroke to review driving test questions and his knowledge of road laws. Instead of helping this person to regain their driving license, the intervention caused anxiety and postponement of the driving rehabilitation process. That experience influenced how and when the occupational therapist introduced the driving assessment process with future patients, as part of the outdoor journey intervention.

An enabler was when therapists encouraged and reassured people with stroke that they were 'ready' to start getting out and about, and obtained a positive response. Therapists reported that people with stroke often sought their approval about going out. Many lacked confidence with their mobility. Therapists also realised that people with stroke needed to be 'ready' to participate in outings.

Second, family members had a large influence on what interventions therapists did, or did not offer. Family members were viewed as being restrictive by several therapists because they discouraged outdoor journeys. Reasons for family members being restrictive included perceived anxiety about the person with stroke falling or getting lost, being unable to manage the distance or having another stroke.

Therapists often wanted to take people with stroke out on escorted journeys to counteract the influence of restrictive family members. Therapists wanted to demonstrate to others, and to themselves, that independent outdoor journeys were safe. However, family members sometimes drove their relative to appointments when a public transport trip had been planned.

Third, the policies of health and community services influenced whether staff provided escorted outdoor journey sessions or not. Risk management policies were most often mentioned. In the following quote, a therapist reflects on how risk management policies in hospital led staff to discourage independent walking by inpatients. However, this advice continued to influence activity levels after hospital discharge. Inpatients followed therapists' advice literally and were anxious about walking unsupervised at home. This advice might explain why some people with stroke found it psychologically difficult to go outdoors alone.

Policies of community services, as well as hospitals could also affect whether people with stroke went outdoors or not. Local community services such as HomeCare were a potential enabler to getting people with stroke out and about. Some services completed grocery shopping early after hospital discharge, or staff took people with stroke with them when they went shopping. More often, however, organisational or insurance policies prohibited service staff from escorting clients to the shops. Staff had to shop alone while the person with stroke remained at home. Thus, their policies could be a barrier to community access training.

In summary, social influences which affected delivery of the outdoor journey intervention included the attitudes, confidence and risk tolerance of people with stroke and their family members. An important barrier to enabling outdoor journeys was the restrictive attitudes of family members. Policies of health and community services also influenced who could provide escorted journeys. Enablers included people with stroke who responded positively to encouragement and reassurance from therapists.

### 2. Beliefs about Capabilities

A second domain affecting therapists' ability to deliver the outdoor journey intervention was their *Beliefs about Capabilities*. This category refers to beliefs about their individual ability, and the ability of their team or family members to provide the intervention. Therapists could be optimistic, pessimistic or ambivalent about delivering the intervention. For some, their attitude changed as a consequence of delivering the outdoor journey intervention; they became more self-assured and confident.

First, we describe the pessimism and negative beliefs about capability. When therapists knew they had to escort people into the community for six sessions, they articulated concerns about spending two or more hours per session with a person, and being alone during these sessions. They knew that public transport training in particular would involve two or more hours of their time.

A novel aspect of the implementation project was that therapists were encouraged to involve assistants on their team, to help share the number of sessions. However, the ability of unqualified therapy assistants was a concern for some therapists. This concern stopped them from delegating sessions to an assistant.

Some therapists were reluctant to suggest outdoor journeys until they had seen a person with stroke for several sessions, and developed a good rapport. The problem with delaying outdoor mobility practice was that by the time therapy finished, typically after six weeks, little or no time had been dedicated to increasing outdoor journeys.

While the interview challenged participants' practice, most rose to the challenge. Therapists reflected positively on how and where they might take a person with stroke to practice road-crossing. They also described in follow-up interviews how they had learned over the year about planning ahead and anticipating problems, to better manage community-based retraining sessions. Other enabling strategies proposed by participating therapists to help increase confidence included having a checklist of items to take on outdoor journeys. This checklist should include medications, a mobile phone, drinking water, an umbrella or sunhat. Items like a mobile phone were already taken on home and community visits by therapists because this was part of area health policy.

To summarise, intervention to help increase outdoor journeys was sometimes limited by therapists' pessimism about their ability to spend two or more hours in the community per therapy session, and beliefs about the abilities of unqualified therapy assistants. An enabler was holding the belief that escorted outdoor sessions could be provided safely by therapists if they anticipated problems and planned ahead well.

### 3. Knowledge and Skills

Knowledge and skills which therapists felt they needed to deliver the intervention fell into one of four categories: (i) components of the outdoor journey intervention itself, (ii) providing advice about return to driving, (iii) about motorised scooters, and (iv) about local transport options. Therapists described a lack of knowledge, skills and confidence in all areas.

Knowledge about the outdoor journey intervention was limited. When therapists were interviewed in 2007, most had not read the seminal publication by Logan and colleagues (2004). Those who had read the publication were still unclear about the components of the intervention, exactly what to do and how often. Most therapists knew the intervention was 'about getting people out into the community' but needed more information about what to actually do. A training workshop was planned to meet this need.

Knowledge about return to driving emerged several times as a gap. Therapists recognised that they lacked accurate information about driving regulations, assessment and rehabilitation. The occupational therapists knew roughly what the local and national guidelines stated, but were still uncertain about how long a person had to wait before attempting an on-road driving assessment, if a person's driving license should be suspended during that time or not, and whether a person had to obtain a Learner Permit.

A third knowledge and skills gap was around motorised scooters - how to assess a person for, prescribe and fund a scooter. There was a general assumption that the local government equipment scheme would not fund scooters. This assumption was incorrect, although the guidelines were strict and approval was rare. Therapists assumed - not unreasonably - that people with stroke would have to buy their own scooter. They reasoned that most people could not afford to buy a scooter themselves, and therefore did not raise this type of mobility device as an option. Therapists were not aware of other funding options which could be pursued. As a consequence of rarely prescribing scooters, therapists recognised a skill gap should they need to help a person with a prescription.

Finally, knowledge about local transport options was another barrier to delivering the outdoor journey intervention. Not only were therapists unfamiliar with local transport options, most did not use buses or trains. They mostly drove to and from work. Therapists needed to learn how local ticketing systems operated, how and where to obtain timetables, and what subsidised transport services were available.

In summary, four categories of skills and knowledge were described by therapists as necessary for implementation of the outdoor journey intervention. Thus, they described skills and knowledge barriers. They needed to know about components of the intervention, return to driving regulations, how to prescribe a motorised scooter, and about local transport options.

### 4. Professional Role Identity

This category refers to whether the outdoor journey intervention was compatible with, and recognised as part of therapists' professional role. Little reference was made to the therapy assistant role. The intervention was considered to be part of the occupational therapy role, yet most participants had not seen occupational therapists offer travel training or take people with stroke to shopping malls. Consequently referrals were not made for these activities. An occupational therapist noted that she asked people with stroke about shopping and banking but did not offer intervention for identified problems. She considered this gap to be part of her role.

Outdoor mobility training did not seem to be covered well by either the occupational therapists or physiotherapists. An occupational therapist considered outdoor mobility training to be part of the physiotherapist's role. Yet this view was not held by all physiotherapists. One of the three physiotherapists interviewed was pessimistic about implementing outdoor mobility training. She was concerned about the role expansion and time involved. If multiple sessions were dedicated to escorted outdoor journeys, time spent on current interventions would be reduced.

Two of the three physiotherapists were more optimistic that they could deliver outdoor mobility training. However, they were all adjusting to the idea of escorting people with stroke and providing practice opportunities in suburban streets, not just in the hospital gym, carpark or grounds. Two of the three therapists did not routinely conduct home or community visits, but held positive attitudes to changing practice, which was an enabler.

Finally, one team decided that physiotherapy and occupational therapy staff would share responsibility for screening people with stroke. Both disciplines and the therapy assistant would deliver the outdoor journey intervention. After several months, role sharing increased, enabling the intervention to be delivered regardless of discipline gaps.

In summary, travel training was not initially perceived to be part of an occupational therapists' role. Yet occupational therapists who were interviewed acknowledged the need to offer intervention if a person with stroke could not get to the shops or the bank. Outdoor mobility training was perceived to be part of a physiotherapists' role, although the time involved in doing 'real-world' training remained an attitudinal barrier.

### 5. Resources

This domain refers to the absence of resources, limited resources, or the unpredictable availability of a resource. Categories of resource included staffing, time, information technology and vehicles. These resources were barriers to delivery of the outdoor journey intervention, although some therapists described strategies (enablers) for overcoming limited resources.

Not unexpectedly, staffing influenced the intervention to be delivered. Absence of an occupational therapist on a community team was initially considered to be an important barrier. The occupational therapist was responsible for taking people with stroke on escorted journeys on buses and trains. Sometimes, a team would have no occupational therapist or only a part-time therapist. One team limited the number of therapy sessions each person with stroke received, to help manage their waiting list and backlog of referrals following staff vacancies.

Large blocks of time - up to three hours - had to be allocated for community-based training. A therapist might leave their office at 9 am, leave the home of a person with stroke at 9.30 am to catch a bus, arrive at a shopping centre or doctor's surgery by 10.00 am, return home by 11.00 am or later, and return to their office by midday. However, therapists expected to spend much less time - about one hour - with most of their patients and clients. They felt guilty spending more than one hour per session. To help manage time, they arranged for colleagues to drop them off or collect them, rather than travel to and from a destination by public transport. These time-saving strategies were an enabler to delivering the outdoor journey intervention.

Limited internet and computer access was the third category of resources which could prevent delivery of the intervention. Without access to these resources, therapists' could not easily provide transport information to people with stroke. The health department could not afford for all health professionals to have internet access, which stopped some therapists - usually junior therapists - from efficiently finding travel information. Instead, they had to wait for other staff members to log them onto the internet.

Vehicles for community visits were the fourth resource which could be in short supply and become a barrier to outings. Therapists anticipated an increase in vehicle bookings and a need for advance planning by staff when delivering the outdoor journey intervention more routinely. Use of taxis was another strategy or enabler that therapists planned to use, to overcome this resource problem.

In summary, therapists identified staffing, time, internet and vehicle access as resources that were in short supply and could be barriers to providing the outdoor journey intervention. However, solutions were also proposed such as role sharing and using taxis.

### 6. Memory and Attention

A fifth domain affecting therapists' ability to deliver the evidence-based intervention was their memory for, and attention to, outdoor mobility and travel. This category also refers to triggers and prompts that reminded therapists to ask people with stroke about outings and travel. If they could remember to ask about these issues, therapists assumed they were more likely to address them during rehabilitation. All professionals who were interviewed felt that changes to their assessment, documentation and case conferencing procedures would help prompt these discussions. By the time of the follow-up interview, one team had made a series of changes to their assessment form and procedures. As anticipated, these changes did help focus their attention on outdoor mobility and travel. The initial assessment form became a prompt and an enabler.

Remembering to ask about outings, transport and driving did not always mean that these topics were documented. Medical record audits raised awareness of this gap. Questioning people with stroke about outings and transport was not enough; they had to remember to document their findings. If a person with stroke did not want to use public transport or be referred for a driving assessment, this information needed to be documented for the benefit of current and future team members:

Placing community access on the case conference agenda was another solution proposed by teams to focus attention on outdoor journeys. Finally, if a person with stroke identified a destination or appointment they had to travel to, this focussed attention. Problems getting to the local doctor because of dependence on family might trigger a discussion about driving or a travel training session:

In summary, existing assessment procedures were a systems barrier. The assessment form did not remind therapists to ask about outdoor mobility and travel. Not surprisingly, it was the assessment procedures and paper form that therapists suggested changing, to help focus attention on the issues. Appointments and destinations could trigger a discussion about outdoor mobility and focussed goals on community participation. Few suggestions were made about prompts to help them remember to deliver the evidence-based intervention, as opposed to asking about outdoor journeys.

### 7. Emotions

This category refers to how therapists felt about delivering (or withholding) the outdoor journey intervention, and their emotional responses. Previous categories have alluded to feelings of guilt because of the time involved in delivering sessions. However, other emotional responses included embarrassment, enjoyment and satisfaction. Such feelings are important to consider because they may inhibit or enable delivery of an intervention.

Feelings of guilt were reported not only about time but also about the nature of the intervention. One occupational therapist felt that an escorted outdoor journey was not 'real' rehabilitation, unlike upper limb or cognitive rehabilitation. Feelings of embarrassment were also reported by the same therapist during escorted journeys. She worried about the privacy and feelings of people with stroke, when neighbours could see them being escorted. However, she also described strategies to minimise embarrassment such as changing the backpack she carried on outings.

Positive feelings were consistently reported by therapists who focussed on increasing outdoor journeys. Therapists felt good about their new role. Feelings of satisfaction and pride resulted when people with stroke appreciated their work. Therapists worried less about cajoling reluctant individuals out into the community when positive feedback was received.

In summary, therapists reported negative and positive emotions towards the outdoor journey intervention. Guilt and embarrassment were reported initially but these feelings were often replaced by enjoyment and satisfaction after delivering the intervention.

## Discussion

This discussion will focus on three key findings. First, therapists provided interventions based partially on what people with stroke expected of them, and the views of family members. This approach is consistent with the principles of evidence-based practice, but may require therapists to negotiate, build confidence and address self-efficacy before clients will venture outdoors. Second, therapists did not know the evidence about community stroke rehabilitation contained in national clinical guidelines, with implications for continuing professional development and knowledge translation. Finally, therapists were uncomfortable offering an intervention which was not part of their traditional or legitimate role. Nonetheless, several professionals were open to professional role revision, which enabled them to accommodate the outdoor journey intervention with existing resources.

### When Clients Decline Evidence-Based Intervention

Evidence-based medicine, and evidence-based practice have been defined as the integration of the best research evidence with clinical expertise and the unique values and circumstances of patients [25]. This process is not as simple as it first appears, as therapists in our study acknowledged. Some people with stroke did not want evidence-based intervention, at least not at the time when it was offered. Not everyone wanted to leave their home and practice crossing roads or travel on a bus. Furthermore, some family members actively discouraged risk-taking. And sometimes both clients and family members expected an intervention which was not supported by strong evidence. These views need to be acknowledged, and negotiation undertaken, if rehabilitation outcomes are to be optimised.

Negotiation with clients around evidence is not unique to the outdoor journey intervention. Other examples from the literature include people with acute back pain who visit their general practitioner expecting to receive a referral for an x-ray, passive manual therapies and advice to rest. Yet clinical guideline recommendations advise against these interventions, and suggest that the person remain active [26]. General practitioners have reported difficulty resisting patient expectations, and many need to practice new consultation scripts before they stop ordering expensive x-rays at a patient's request [27]. Therapists in our study did not appear to have difficulty changing their intervention practices once they knew about the evidence.

Innovative strategies will be required if occupational therapists and physiotherapists are to negotiate around family values, and overcome risk intolerance. Other recent data collected from people with stroke substantiates the influence that family members can have on mobility and travel training by therapists (Barnsley, McCluskey & Middleton, What people say about travelling outdoors after a stroke: A qualitative study, submitted). In that qualitative study, 19 people with stroke received the outdoor journey intervention, and talked about their lack of confidence crossing roads and walking over rough ground, as well as fear associated with bus travel. Family members who did not allow travel on public transport and encouraged dependence on family car travel compounded this lack of confidence. People who are discouraged from walking and traveling outdoors can lose mobility gained during their inpatient stay. They risk becoming physically inactive and unnecessarily socially isolated. Family burden may increase. All these secondary consequences of stroke are avoidable, at least in theory.

Strategies which may assist therapists to negotiate around these challenges include the use of educational materials such as written narratives or videotaped vignettes of people with stroke who travel outdoors. These educational materials could be viewed in hospital prior to discharge or at home. Materials could address concerns about risk and safety, personal goal setting, and how people with stroke overcome their lack of confidence with practice and escorted journeys. Benefits for family members, such as reduced burden resulting from increased independence could also be recorded for educational purposes.

### Building Confidence and Self-Efficacy

It is ultimately the person with stroke who makes the final decision about intervention. Some people will choose to adopt a sedentary lifestyle and travel infrequently into the community. While information can be provided about risk factors, particularly physical inactivity, the person's decision needs to be respected. In such situations, therapists cannot, and should not, impose an intervention onto people, no matter how large the potential benefit. Unfortunately, we did not explore therapists' attitudes and responses to this dilemma in-depth during the interviews. While therapists often delayed the intervention by a few weeks, until the person with stroke was 'ready', they did not describe any strategies for addressing fear and lack of confidence.

Loss of confidence featured prominently as a barrier to therapists successfully delivering the outdoor journey intervention. Self-efficacy is a psychological construct similar to perceived confidence which originates from Bandura, and refers to an individuals' belief in their own ability to change behaviours such as walking outdoors or resuming driving. Self-efficacy is thought to influence whether a person will attempt an activity and persevere when they encounter barriers. Studies exploring self-efficacy after stroke are limited, but examples do exist [28]. If a person with stroke is concerned about falling when walking to the postbox, for example, a self-management programme where they set personal goals and targets may be helpful. Some of these goals may involve community participation and confidence building. Therapists could obtain before-and-after measures using instruments such as the Stroke Self-Efficacy Questionnaire or SSEQ [29]. The SSEQ would enable therapists to identify people with stroke who have low self-efficacy and are reluctant to travel outdoors. Therapists can then help the person to set goals, and target skills and activities of importance to that person.

### Keeping Up-to-Date with the Evidence

Lack of knowledge about the evidence from national clinical guidelines should not have been a surprise to us, but it was. When these therapists were interviewed in 2007, none were aware of the original trial [11], the nature of the intervention [12] or the relevant guideline recommendation [13]. This lack of knowledge existed despite mailed dissemination of the clinical guidelines, a national workshop being run about guideline implementation, and publication of a critically appraised paper about the original trial [30]. These printed educational materials and the workshop were provided as far back as 2005 to aid knowledge transfer.

A key challenge for professionals who try to keep up to date with evidence is their mixed caseload. In addition to seeing people with stroke, many see people with amputations, dementia and hip fractures. Evidence-based interventions for some of these conditions have also been recommended in other recent systematic reviews [31,32] and will subsequently be included in guideline recommendations. Allied health professionals, like general practitioners, are often expected to keep up to date with evidence across many practice fields. A team strategy is needed if knowledge is to remain current. One strategy might be for each team member to assume responsibility for checking best evidence and attending conferences about particular conditions. Another possible strategy is the establishment of a journal club where recent systematic reviews and guidelines are appraised. Publications can be identified using free web-based resources such as the Cochrane database, the Physiotherapy Evidence Database (available at http://www.pedro.org.au) and the OTseeker database for occupational therapists (available at http://www.otseeker.com). Each resource contains evidence about effective interventions relevant to allied health professionals.

Poor searching and information literacy skills may be another reason why therapists did not know about the evidence. Therapists reported computer and internet access as a barrier to obtaining transport information. This resource problem may have been a barrier to accessing websites such as those previously mentioned, and obtaining published journal articles. Unfortunately, these issues were not discussed in-depth during the interviews, nor were strategies for keeping up to date. Future studies should aim to explore reasons for lack of knowledge in more depth. While one of the authors has previously identified poor internet access as a barrier to evidence-based practice in this local population [33], data were collected in 2000 using a self-report survey. More recent studies have explored how, when and where Australian health professionals conduct their searches [34-36], but do not tell us about local resources and barriers.

### Professional Roles: A Barrier and an Enabler

The original trial intervention had been delivered exclusively by occupational therapists. Several team members whom we interviewed recognised the need to expand team roles. Over time, one team began sharing responsibility for delivering multiple outdoor journey sessions. Neither the occupational therapists nor physiotherapists felt they could deliver six or more sessions alone with existing resources, nor were they protective of their roles. While both professions had to expand their roles and spend more time outdoors with clients, one team split the sessions based on expertise. The physiotherapist assessed and practiced overground walking, road crossing, and safety on slopes and stairs. The occupational therapist assessed and practiced bus and train travel, walking while carrying items in shopping malls, and driving-related tasks. The therapy assistant supported therapy sessions by providing additional escorted journeys.

Substitution of tasks from one professional to another is an organisational change known as role revision [37] or expansion. This change involves the transfer or supplementation of tasks, and may involve new or existing team members. Most research to date has investigated the effect on patient care of nursing professionals or pharmacists replacing doctors, or the effect of role sharing [38,39]. The current study suggests that professional role revision and expansion in community rehabilitation may have potential benefits for people with stroke. No robust research to date has investigated role changes in allied health. The effect of this organisational change in community rehabilitation, and the use of allied health therapy assistants warrant further research. In particular, a study is needed which examines the effect of allied health therapy assistants on patient outcomes and the cost of service delivery.

### Barriers and Enablers Not Reported

Unreported barriers and enablers are just as important to consider as those which are reported. Unreported factors tell service managers what is already working well within an organisation or team. First, none of the professionals interviewed challenged the evidence. No-one disputed the quality of study methods or refused outright to deliver the outdoor journey intervention based on strong beliefs. Non-acceptance of evidence has been proposed as one reason why research may not be implemented, and why not all clients receive the benefits [40]. These professionals seemed to have a good understanding of evidence and the importance of randomised controlled trials; however, no objective measures were obtained of their competence, knowledge or skills, for example using the Adapted Fresno Test of Competence in Evidence-Based Practice [41].

Second, lack of leadership and management support was not raised as a problem. It is possible that participants felt unable to raise concerns about management because the interviewer (the first author) had direct links with senior management in the organisation. Finally, of importance to implementation is the fact that none of the barriers seemed insurmountable to participants. None of the barriers raised were too large to overcome.

### Maintaining the Fidelity of the Original Intervention

Even when these occupational therapists and physiotherapists in our study knew about the effectiveness of the outdoor journey intervention, they still did not know what to do, nor how to deliver the therapy sessions. The original triallists have published a helpful description of the outdoor journey intervention [12]. However, no protocol existed for upgrading outdoor walking (eg distances and terrain), stair climbing, bus or train travel which were the core 'deliverables' of the intervention. No safety protocols were available to address concerns about patient safety and risk management. These protocols had to be written and tested by the first author [42]. Without such protocols, the fidelity of an intervention is questionable.

Research is now being conducted by others to measure the fidelity of complex interventions [43-45]. Fidelity scales measure adherence to critical components of a particular practice such as screening, assessment, instruction and training of clients as per written protocols. For example, a rating of five might indicate full adherence while one indicates no adherence. Written protocols not only address the skills, knowledge and capability barriers reported by participants, they also help to promote program fidelity. The authors of the current study have been in regular contact with the original triallists while writing the intervention and safety protocols. Measuring adherence to these protocols has not yet been attempted.

### Study Limitations

As with all studies, this research had some limitations. First, the qualitative design involved two teams from one city, in one country. Barriers reported by other teams will vary. However, this process of identifying local barriers is arguably the only way to identify unique factors which will inform a tailored intervention for particular teams. Some of the barriers raised by our participants can be anticipated when time-intensive community-based interventions are being implemented elsewhere.

A second limitation - but also a strength - was the use of a guiding theoretical framework during analysis, and during the second round of interviews. Use of this theory may have prevented categories from emerging which did not 'fit' the theory presented by Michie and colleagues (2005); however, we were careful to complete our initial coding prior to using the theory, and to continue generating 'free' unrestricted categories throughout analysis.

### Contribution to Knowledge

The current study adds to knowledge about implementation of evidence and knowledge transfer in several ways. First, we were able to describe perceived barriers to practice change, before and while professionals implemented the outdoor journey intervention. Importantly, this information will be used to plan a larger cluster randomised trial involving multiple teams and patient participants. Second, we have demonstrated the utility of the theory described by Michie and colleagues, using their prompt questions to aid data collection and category labels to aid qualitative analysis.

### Implications for Practice and Research

There are several implications for practice. First, the study provides a real example of how client values can and do impact on the delivery of evidence-based practice. A range of strategies are proposed for managing differences in expectations around treatment. Some people with stroke will continue to decline interventions, and their views need to be respected. For others, time may need to be spent building confidence and self-efficacy. Vignettes and narratives of people talking about their personal goals, gaining confidence and travelling outdoors after a stroke may be useful, as well as instruments that measure self-efficacy. Educational resources could also include the experiences of family members as they learn to tolerate risk.

A second practice implication is that many community teams will need strategies for staying up to date with evidence across multiple caseloads. Examples of strategies include giving team members responsibility for different health conditions, and evidence-based journal clubs which address different health conditions. Also when introducing a new evidence-based intervention, education is likely to be needed about the intervention protocol, use of special equipment, skill development and so forth.

Third, although different teams may identify unique resource problems, many can be resolved locally. Staffing gaps were addressed. Difficulties accessing computers and on-line timetables were also overcome. Finally, role sharing by team members, and use of allied health assistants may have the potential to address multiple barriers and enable delivery of time-intensive interventions, such as the outdoor journey intervention.

Future research should explore the effect of using allied health assistants to substitute for or supplement qualified professionals, in hospital and community settings. Patient outcomes and the impact on service costs should be investigated as a result of this type of organisational change. Another research implication is the potential value of using a theory of behaviour change to guide and inform qualitative data analysis. The theory described by Michie and colleagues (2005) helped us to streamline the interview schedule and data analysis. Domains in the theory helped us to explore possible barriers such as role identity; role sharing later became an enabler to implementation.

## Conclusions

Knowledge translation is an important final step in the process of evidence-based practice. The process described in this paper allowed us to identify barriers which became the focus of a tailored behaviour change intervention, to help translate knowledge into practice. The tailored intervention involved education about the outdoor journey intervention, medical record audits followed by feedback and reminders. The process and barriers may help other researchers who wish to translate a complex intervention into practice.

## Competing interests

The authors declare that they have no competing interests.

## Authors' contributions

The first author conceptualised and planned the study, collected and analysed the data and drafted the manuscript. The second author advised on study design, and writing of the manuscript. Both authors read and approved the final manuscript.

## Pre-publication history

The pre-publication history for this paper can be accessed here:

http://www.biomedcentral.com/1472-6963/10/18/prepub

## Supplementary Material

Additional file 1 ***Barriers to delivering the outdoor journey intervention with multiple quotations***. This file contains additional quotations from participants. Click here for file
