# Supplementary material for: Delivering an evidence-based outdoor journey intervention to people with stroke: Barriers and enablers experienced by community rehabilitation teams
Source: BMC Health Serv Res. 2010 Jan 19;10:18. doi: 10.1186/1472-6963-10-18 (PMC2821384; doi:10.1186/1472-6963-10-18)
Supplement: Additional file 1 — Barriers to delivering the outdoor journey intervention with multiple quotations. This file contains additional quotations from participants. [file 1472-6963-10-18-S1.PDF]

Additional File 1. Barriers to delivering the outdoor journey intervention with multiple quotations ^

Social influences: Unhelpful influences from people with stroke, family members, health professionals and community service providers that discouraged delivery of the outdoor journey intervention. Unhelpful influences included expectations about intervention, low tolerance for risk and restrictive organisational policies.

From people with stroke: Some people are more focussed on getting their arm to work again 'I just want my arm to work'…[so] trying to identify goals different from that can be hard. Usually they've been told 'We're referring you for upper limb therapy' and they become very focussed on that 'I want to move my arm again'. ..they don't want to ….look at …getting out and about. (OT1)

From family members: Last week they had an opportunity of catching the train into the sessions. But the wife called their son to tell him to take the day off work to take him…she says 'No, he won't be able to do it'. (SP1)

From policy: The policy is …that they won't take someone out in the car with them unless it's specifically for transport (eg to transport a person from home to an appointment). But if the person needs shopping, the worker goes out instead of the patient….it's encouraging the patient to be housebound and not go into the community. (OT3).

Belief about capabilities: Unhelpful, pessimistic or ambivalent beliefs and attitudes about the capability of an individual professional, family member, or the team to provide the outdoor journey intervention.

…there's concern about [therapy assistants] having the skills to assess and analyse the situation…..Would everything [ie problem areas] be picked up by somebody who's not trained? Can we have somebody who's not fully trained doing interventions and sessions? (OTM1)

Maybe [it's] partly to do with not having done this before…for people… gosh …(thinks)…how do I get people on buses? And time wise….[how do I manage]? (OT2)

Knowledge and skills: Knowledge and skills which professionals felt they lacked, which related to; (i) the intervention itself; (ii) return to driving, (iii) motorised scooters and (iv) local transport options.

(i) Knowing about the intervention: Is it just public transport? Or is it…using the [subsidised] transport services? (OT1)

(ii) Giving advice about return to driving: One thing that the staff felt they wanted was information…around driving and what the guidelines say. Where can we access information to help them [people with stroke] for the knowledge part of the driving assessment? [Staff] awareness of what actually happens in an occupational therapy driving assessment is limited….how that's separate from the RTA [Roads and Traffic Authority] test, and sitting at a computer… and going out with a driving instructor? (OT3)

(iii) Prescribing a motorised scooter: *For the occupational therapists that have come through our service, I think the whole scooter thing is still something they are nervous about. They… don't get a lot of exposure …I've never done a scooter prescription, mainly because I've worked in a hospital and not in the community (OT3)*

(iv) Knowledge of local transport options: I have no idea about public transport in this area … identifying bus routes, the cost of it, where you catch buses from. In the past I've had to rely on the patients to tell me where the bus stop is, and where the bus routes are. Same thing if you're going catch a train with a patient… The train timetable… I have no idea. (OT1)

Professional role identity: Unhelpful attitudes and beliefs about professional roles, and routines which presented a barrier to delivery of the intervention. Includes professionals who were pessimistic or ambivalent about role change.

You can only do so much...if you put all your eggs in one basket, then the others are empty… the only way we can do something is if…..[we have] resources [or] if we change something else. Maybe [another named service] should do more. (PT2)

I think a lot more people COULD benefit from [this type of] occupational therapy than actually have it. That's the first barrier. We as non-occupational therapists wouldn't normally think to refer to [them] for this aspect of care…. much of the role seems to have been about upper limb training and maybe some cognitive training. (PT1)

Resources: The absence of resources, limited resources or unpredictable resources on which the intervention depended. There were four categories of resources: (i) staff; (ii); time; (ii) information technology; and (iv) vehicles.

(i) Staffing: At the moment we're working together with speech and physio… [we're] really wanting to work together to be able to do this community access. [but] ….it's hard to keep access [open for patients]. ….it's a lot to do with staff - or lack of staff (OT2)

(ii) Time: [I aim to] cut [the time] in half…[when I go out on] public transport…I will drive somewhere, then we do the shopping, then we come back because I cannot afford more than two hours a trip (OT4)

(iii) Information technology: To get a bus timetable and transport information …I'm lucky now I have access to the internet, but before I didn't, and you'd have to get someone else to log you on, which can make it difficult (OT1)

(iv) Vehicles: Getting cars has been a huge issue in the past. We don't have a [dedicated] car for that position. If there [wasn't] a car…it [would require] a bit more planning and approval (OTM1)

Memory and attention: Failure or difficulty remembering to ask about, assess, document or address outdoor mobility and travel because of current systems, procedures or habits.

Sometimes I might bring it [driving referral] up in discussion but I might forget when it comes time to document (OT1).

I only document that we are going by bus or by car. I never even thought to document where we are going (OT2).

Emotions: Negative or uncomfortable emotions when thinking about, or delivering the intervention.

If I'm out with a client for three hours or so, there will be a feeling of guilt…it's a bit like a social outing…I know we're training community access but perhaps a feeling of guilt that you're gone for so long, with one person and you're out in the community (OT2)

I don't take a backpack...a little backpack is OK but the huge backpacks with your ID badge, your blue uniform…people automatically think we're nurses…neighbours are having a look…'What's going on here?'. Do they think I'm a psych[iatric] nurse? I would probably feel …uncomfortable about…not so much for me but for the person I'm taking out. (OT2)

Notes. ^ The following abbreviations are used after quotations, to represent each allied health profession: occupational therapy (OT); physiotherapy (PT); speech pathology (SP); and social work (SW). Numbers (eg OT1, PT2) correspond to de-identified individual professionals as summarised in Table 1.
